# Supplementary material for: Pregnancy Recommendations Solely Based on Preclinical Evidence Should Be Integrated with Real-World Evidence: A Disproportionality Analysis of Certolizumab and Other TNF-Alpha Inhibitors Used in Pregnant Patients with Psoriasis
Source: Pharmaceuticals (Basel). 2024 Jul 7;17(7):904. doi: 10.3390/ph17070904 (PMC11279583; doi:10.3390/ph17070904)
Supplement: Supplementary file 1 [file pharmaceuticals-17-00904-s001.zip › Supplementary Table S1.pdf]

Table S1. Preferred Terms included in our analysis on pregnant-outcomes and fetal/neonatal disorders.

**smq_name**	**pt_name**
Foetal disorders (SMQ)	Alpha 1 foetoprotein abnormal
Foetal disorders (SMQ)	Alpha 1 foetoprotein amniotic fluid abnormal
Foetal disorders (SMQ)	Alpha 1 foetoprotein amniotic fluid increased
Foetal disorders (SMQ)	Alpha 1 foetoprotein decreased
Foetal disorders (SMQ)	Alpha 1 foetoprotein increased
Foetal disorders (SMQ)	Amniocentesis abnormal
Foetal disorders (SMQ)	Amnioscopy abnormal
Foetal disorders (SMQ)	Bradycardia foetal
Foetal disorders (SMQ)	Cerebral infarction foetal
Foetal disorders (SMQ)	Diethylstilboestrol syndrome
Foetal disorders (SMQ)	Ductus arteriosus stenosis foetal
Foetal disorders (SMQ)	Erythroblastosis foetalis
Foetal disorders (SMQ)	Foetal alcohol syndrome
Foetal disorders (SMQ)	Foetal arrhythmia
Foetal disorders (SMQ)	Foetal damage
Foetal disorders (SMQ)	Foetal distress syndrome
Foetal disorders (SMQ)	Foetal malnutrition
Foetal disorders (SMQ)	Foetal-maternal haemorrhage
Foetal disorders (SMQ)	Hydrops foetalis
Foetal disorders (SMQ)	Hypopituitarism foetal
Foetal disorders (SMQ)	Maternal death affecting foetus
Foetal disorders (SMQ)	Maternal drugs affecting foetus
Foetal disorders (SMQ)	Maternal hypertension affecting foetus
Foetal disorders (SMQ)	Meconium increased
Foetal disorders (SMQ)	Oligohydramnios
Foetal disorders (SMQ)	Polyhydramnios
Foetal disorders (SMQ)	Tachycardia foetal
Foetal disorders (SMQ)	Ultrasound antenatal screen abnormal
Foetal disorders (SMQ)	Umbilical cord around neck
Foetal disorders (SMQ)	Umbilical cord compression
Foetal disorders (SMQ)	Umbilical cord prolapse
Foetal disorders (SMQ)	Umbilical cord short
Foetal disorders (SMQ)	Umbilical cord vascular disorder
Foetal disorders (SMQ)	Virilism foetal
Foetal disorders (SMQ)	Biopsy chorionic villous abnormal

Foetal disorders (SMQ)	Umbilical cord abnormality
Foetal disorders (SMQ)	Cerebral haemorrhage foetal
Foetal disorders (SMQ)	Foetal acidosis
Foetal disorders (SMQ)	Paternal drugs affecting foetus
Foetal disorders (SMQ)	Angiotensin converting enzyme inhibitor foetopathy
Foetal disorders (SMQ)	Meconium in amniotic fluid
Foetal disorders (SMQ)	Foetal heart rate decreased
Foetal disorders (SMQ)	Foetal heart rate increased
Foetal disorders (SMQ)	Foetal heart rate abnormal
Foetal disorders (SMQ)	Amniotic infection syndrome of Blane
Foetal disorders (SMQ)	Foetal warfarin syndrome
Foetal disorders (SMQ)	Amniorrhexis
Foetal disorders (SMQ)	Foetal cystic hygroma
Foetal disorders (SMQ)	Foetal cardiac disorder
Foetal disorders (SMQ)	Anaesthetic complication foetal
Foetal disorders (SMQ)	Foetal cerebrovascular disorder
Foetal disorders (SMQ)	Radiation injury affecting foetus
Foetal disorders (SMQ)	Foetal macrosomia
Foetal disorders (SMQ)	Intrauterine infection
Foetal disorders (SMQ)	Meconium stain
Foetal disorders (SMQ)	Foetal therapeutic procedure
Foetal disorders (SMQ)	Maternal condition affecting foetus
Foetal disorders (SMQ)	Meconium peritonitis
Foetal disorders (SMQ)	Biopsy foetal abnormal
Foetal disorders (SMQ)	Diabetic foetopathy
Foetal disorders (SMQ)	Alpha 1 foetoprotein amniotic fluid decreased
Foetal disorders (SMQ)	Foetal malformation
Foetal disorders (SMQ)	Amniotic cavity disorder
Foetal disorders (SMQ)	Amniotic cavity infection
Foetal disorders (SMQ)	Foetal disorder
Foetal disorders (SMQ)	Foetal heart rate disorder
Foetal disorders (SMQ)	Haemorrhage foetal
Foetal disorders (SMQ)	Amniotic fluid volume decreased
Foetal disorders (SMQ)	Amniotic fluid volume increased
Foetal disorders (SMQ)	Foetal chromosome abnormality
Foetal disorders (SMQ)	Omphalorrhexis
Foetal disorders (SMQ)	Umbilical cord haemorrhage
Foetal disorders (SMQ)	Amniotic fluid erythropoietin level increased
Foetal disorders (SMQ)	Cordocentesis
Foetal disorders (SMQ)	Amniorrhoea

Foetal disorders (SMQ)	Foetal anticonvulsant syndrome
Foetal disorders (SMQ)	Amniotic fluid index abnormal
Foetal disorders (SMQ)	Vesicoamniotic shunt
Foetal disorders (SMQ)	Foetal hypokinesia
Foetal disorders (SMQ)	Mirror syndrome
Foetal disorders (SMQ)	Discordant twin
Foetal disorders (SMQ)	Prenatal screening test abnormal
Foetal disorders (SMQ)	Foetal growth restriction
Foetal disorders (SMQ)	Foetal methotrexate syndrome
Foetal disorders (SMQ)	Thalidomide embryopathy
Foetal disorders (SMQ)	Foetal monitoring abnormal
Foetal disorders (SMQ)	Foetal non-stress test abnormal
Foetal disorders (SMQ)	Umbilical cord thrombosis
Foetal disorders (SMQ)	Chronic villitis of unknown etiology
Foetal disorders (SMQ)	Gestational age test abnormal
Foetal disorders (SMQ)	Twin reversed arterial perfusion sequence malformation
Foetal disorders (SMQ)	Foetal megacystis
Foetal disorders (SMQ)	Hypocalvaria
Foetal disorders (SMQ)	Foetal retinoid syndrome
Foetal disorders (SMQ)	Foetal heart rate deceleration abnormality
Foetal disorders (SMQ)	Baseline foetal heart rate variability disorder
Foetal disorders (SMQ)	Nonreassuring foetal heart rate pattern
Foetal disorders (SMQ)	Foetal heart rate acceleration abnormality
Foetal disorders (SMQ)	Sinusoidal foetal heart rate pattern
Foetal disorders (SMQ)	Umbilical cord occlusion
Foetal disorders (SMQ)	Foetal tachyarrhythmia
Foetal disorders (SMQ)	Foetal movement disorder
Foetal disorders (SMQ)	Foetal anaemia
Foetal disorders (SMQ)	Ultrasound foetal abnormal
Foetal disorders (SMQ)	Foetal gastrointestinal tract imaging abnormal
Foetal disorders (SMQ)	Foetal musculoskeletal imaging abnormal
Foetal disorders (SMQ)	Foetal renal imaging abnormal
Foetal disorders (SMQ)	Foetal growth abnormality
Foetal disorders (SMQ)	Foetal compartment fluid collection
Foetal disorders (SMQ)	Foetal biophysical profile score equivocal

Foetal disorders (SMQ)	Foetal biophysical profile score abnormal
Foetal disorders (SMQ)	Foetal renal impairment
Foetal disorders (SMQ)	Umbilical cord cyst
Foetal disorders (SMQ)	Foetal heart rate indeterminate
Foetal disorders (SMQ)	Foetal surgery
Foetal disorders (SMQ)	Enlarged foetal cisterna magna
Foetal disorders (SMQ)	Foetal cardiac arrest
Foetal disorders (SMQ)	Umbilical artery vascular resistance increased
Foetal disorders (SMQ)	Foetal vascular malperfusion
Foetal disorders (SMQ)	Increased foetal movements
Foetal disorders (SMQ)	Foetal tobacco syndrome
Foetal disorders (SMQ)	Foetal cardiac function test abnormal
Foetal disorders (SMQ)	Foetal brain scan abnormal
Foetal disorders (SMQ)	Drug administered to foetus
Foetal disorders (SMQ)	Foetal alcohol spectrum disorder
Foetal disorders (SMQ)	Gestational alloimmune liver disease
Foetal disorders (SMQ)	Maternal-foetal therapy
Foetal disorders (SMQ)	Foetal posterior cerebral artery
Neonatal disorders (SMQ)	ABO incompatibility
Neonatal disorders (SMQ)	Acne infantile
Neonatal disorders (SMQ)	Adrenocortical insufficiency neonatal
Neonatal disorders (SMQ)	Agitation neonatal
Neonatal disorders (SMQ)	Anaemia neonatal
Neonatal disorders (SMQ)	Apgar score low
Neonatal disorders (SMQ)	Arrhythmia neonatal
Neonatal disorders (SMQ)	Atelectasis neonatal
Neonatal disorders (SMQ)	Atrial septal defect repair
Neonatal disorders (SMQ)	Birth trauma
Neonatal disorders (SMQ)	Blood loss anaemia neonatal
Neonatal disorders (SMQ)	Breast engorgement in newborn
Neonatal disorders (SMQ)	Bronchopulmonary dysplasia
Neonatal disorders (SMQ)	Cardiac arrest neonatal
Neonatal disorders (SMQ)	Cardio-respiratory arrest neonatal
Neonatal disorders (SMQ)	Cephalhaematoma
Neonatal disorders (SMQ)	Cerebral haemorrhage neonatal
Neonatal disorders (SMQ)	Circulatory failure neonatal
Neonatal disorders (SMQ)	Cleft palate repair
Neonatal disorders (SMQ)	Coagulation disorder neonatal
Neonatal disorders (SMQ)	Collodion baby
Neonatal disorders (SMQ)	Coxsackie viral disease of the newborn

Neonatal disorders (SMQ)	Cranial nerve injury secondary to birth trauma
Neonatal disorders (SMQ)	Cyanosis neonatal
Neonatal disorders (SMQ)	Death neonatal
Neonatal disorders (SMQ)	Diarrhoea infectious neonatal
Neonatal disorders (SMQ)	Disseminated intravascular coagulation in newborn
Neonatal disorders (SMQ)	Drug withdrawal syndrome neonatal
Neonatal disorders (SMQ)	Dyskinesia neonatal
Neonatal disorders (SMQ)	Eczema infantile
Neonatal disorders (SMQ)	Encephalopathy neonatal
Neonatal disorders (SMQ)	Endocardial cushion defect repair
Neonatal disorders (SMQ)	Extraocular retinoblastoma
Neonatal disorders (SMQ)	Facial nerve injury due to birth trauma
Neonatal disorders (SMQ)	Failure to thrive
Neonatal disorders (SMQ)	Fever neonatal
Neonatal disorders (SMQ)	Fontanelle bulging
Neonatal disorders (SMQ)	Fracture of clavicle due to birth trauma
Neonatal disorders (SMQ)	Gangrene neonatal
Neonatal disorders (SMQ)	Granulocytopenia neonatal
Neonatal disorders (SMQ)	Grey syndrome neonatal
Neonatal disorders (SMQ)	Haemorrhagic disease of newborn
Neonatal disorders (SMQ)	Hepatitis neonatal
Neonatal disorders (SMQ)	Hepatocellular damage neonatal
Neonatal disorders (SMQ)	Hepatosplenomegaly neonatal
Neonatal disorders (SMQ)	Hyperbilirubinaemia neonatal
Neonatal disorders (SMQ)	Hyperkinesia neonatal
Neonatal disorders (SMQ)	Hypoglycaemia neonatal
Neonatal disorders (SMQ)	Hypokinesia neonatal
Neonatal disorders (SMQ)	Hypotonia neonatal
Neonatal disorders (SMQ)	Hypoventilation neonatal
Neonatal disorders (SMQ)	Immature respiratory system
Neonatal disorders (SMQ)	Infantile colic
Neonatal disorders (SMQ)	Infantile scurvy
Neonatal disorders (SMQ)	Infantile spasms
Neonatal disorders (SMQ)	Injury to brachial plexus due to birth trauma
Neonatal disorders (SMQ)	Injury to spinal cord secondary to birth trauma
Neonatal disorders (SMQ)	Intraventricular haemorrhage neonatal
Neonatal disorders (SMQ)	Isoimmune haemolytic disease
Neonatal disorders (SMQ)	Jaundice neonatal

Neonatal disorders (SMQ)	Kernicterus
Neonatal disorders (SMQ)	Large for dates baby
Neonatal disorders (SMQ)	Late metabolic acidosis of newborn
Neonatal disorders (SMQ)	Lymphocytopenia neonatal
Neonatal disorders (SMQ)	Meconium ileus
Neonatal disorders (SMQ)	Meconium increased
Neonatal disorders (SMQ)	Myasthenia gravis neonatal
Neonatal disorders (SMQ)	Neonatal alveolar aeration excessive
Neonatal disorders (SMQ)	Neonatal anoxia
Neonatal disorders (SMQ)	Neonatal asphyxia
Neonatal disorders (SMQ)	Neonatal candida infection
Neonatal disorders (SMQ)	Neonatal diabetes mellitus
Neonatal disorders (SMQ)	Neonatal disorder
Neonatal disorders (SMQ)	Neonatal exchange blood transfusion
Neonatal disorders (SMQ)	Neonatal infective mastitis
Neonatal disorders (SMQ)	Neonatal intestinal obstruction
Neonatal disorders (SMQ)	Neonatal respiratory acidosis
Neonatal disorders (SMQ)	Neonatal respiratory alkalosis
Neonatal disorders (SMQ)	Neonatal respiratory arrest
Neonatal disorders (SMQ)	Neonatal respiratory depression
Neonatal disorders (SMQ)	Neonatal respiratory distress
Neonatal disorders (SMQ)	Neonatal respiratory distress syndrome
Neonatal disorders (SMQ)	Neonatal respiratory failure
Neonatal disorders (SMQ)	Neonatal thyrotoxicosis
Neonatal disorders (SMQ)	Neuroblastoma
Neonatal disorders (SMQ)	Neutropenia neonatal
Neonatal disorders (SMQ)	Omphalitis
Neonatal disorders (SMQ)	Ophthalmia neonatorum
Neonatal disorders (SMQ)	Patent ductus arteriosus repair
Neonatal disorders (SMQ)	Postmature baby
Neonatal disorders (SMQ)	Premature baby
Neonatal disorders (SMQ)	Purpura neonatal
Neonatal disorders (SMQ)	Pyloromyotomy
Neonatal disorders (SMQ)	Rash neonatal
Neonatal disorders (SMQ)	Renal failure neonatal
Neonatal disorders (SMQ)	Repair of imperforate rectum
Neonatal disorders (SMQ)	Respiratory tract haemorrhage neonatal
Neonatal disorders (SMQ)	Retinopathy of prematurity
Neonatal disorders (SMQ)	Sepsis neonatal
Neonatal disorders (SMQ)	Small for dates baby
Neonatal disorders (SMQ)	Somnolence neonatal

Neonatal disorders (SMQ)	Subarachnoid haemorrhage neonatal
Neonatal disorders (SMQ)	Subdural haemorrhage neonatal
Neonatal disorders (SMQ)	Sudden infant death syndrome
Neonatal disorders (SMQ)	Tetanus neonatorum
Neonatal disorders (SMQ)	Thrombocytopenia neonatal
Neonatal disorders (SMQ)	Thrombophlebitis neonatal
Neonatal disorders (SMQ)	Transient hypogammaglobulinaemia of infancy
Neonatal disorders (SMQ)	Transient tachypnoea of the newborn
Neonatal disorders (SMQ)	Tremor neonatal
Neonatal disorders (SMQ)	Umbilical sepsis
Neonatal disorders (SMQ)	Urinary tract infection neonatal
Neonatal disorders (SMQ)	Ventricular septal defect repair
Neonatal disorders (SMQ)	Vision abnormal neonatal
Neonatal disorders (SMQ)	Weight decrease neonatal
Neonatal disorders (SMQ)	Hypertonia neonatal
Neonatal disorders (SMQ)	Immature larynx
Neonatal disorders (SMQ)	Neonatal hyponatraemia
Neonatal disorders (SMQ)	Neonatal hypotension
Neonatal disorders (SMQ)	Herpes simplex virus conjunctivitis neonatal
Neonatal disorders (SMQ)	Neonatal tachycardia
Neonatal disorders (SMQ)	Renal impairment neonatal
Neonatal disorders (SMQ)	Melaena neonatal
Neonatal disorders (SMQ)	Neonatal anuria
Neonatal disorders (SMQ)	Peripheral oedema neonatal
Neonatal disorders (SMQ)	Neonatal cardiac failure
Neonatal disorders (SMQ)	Hypertension neonatal
Neonatal disorders (SMQ)	Neonatal neuroblastoma
Neonatal disorders (SMQ)	Craniosynostosis
Neonatal disorders (SMQ)	Neonatal hepatomegaly
Neonatal disorders (SMQ)	Intoxication by breast feeding
Neonatal disorders (SMQ)	Hypothermia neonatal
Neonatal disorders (SMQ)	Neonatal hypoxia
Neonatal disorders (SMQ)	Poor weight gain neonatal
Neonatal disorders (SMQ)	Cardiomyopathy neonatal
Neonatal disorders (SMQ)	Haemolysis neonatal
Neonatal disorders (SMQ)	Hygroma colli
Neonatal disorders (SMQ)	Neonatal oversedation
Neonatal disorders (SMQ)	Neonatal multi-organ failure
Neonatal disorders (SMQ)	Pulmonary oedema neonatal
Neonatal disorders (SMQ)	Leukopenia neonatal
Neonatal disorders (SMQ)	Floppy infant

Neonatal disorders (SMQ)	Meconium abnormal
Neonatal disorders (SMQ)	High-pitched crying
Neonatal disorders (SMQ)	Periventricular leukomalacia
Neonatal disorders (SMQ)	Systemic-pulmonary artery shunt
Neonatal disorders (SMQ)	Anaesthetic complication neonatal
Neonatal disorders (SMQ)	Fontanelle depressed
Neonatal disorders (SMQ)	Neonatal pneumonia
Neonatal disorders (SMQ)	Meningoencephalitis herpes simplex neonatal
Neonatal disorders (SMQ)	Neonatal mucocutaneous herpes simplex
Neonatal disorders (SMQ)	Group B streptococcus neonatal sepsis
Neonatal disorders (SMQ)	Pulmonary dysmaturity syndrome
Neonatal disorders (SMQ)	Inclusion conjunctivitis neonatal
Neonatal disorders (SMQ)	Delayed closure of cranial sutures
Neonatal disorders (SMQ)	Delayed fontanelle closure
Neonatal disorders (SMQ)	Neonatal respiratory distress syndrome prophylaxis
Neonatal disorders (SMQ)	Necrotising enterocolitis neonatal
Neonatal disorders (SMQ)	Erythema toxicum neonatorum
Neonatal disorders (SMQ)	Caput succedaneum
Neonatal disorders (SMQ)	Perinatal brain damage
Neonatal disorders (SMQ)	Bradycardia neonatal
Neonatal disorders (SMQ)	Neonatal cholestasis
Neonatal disorders (SMQ)	Neonatal warming therapy
Neonatal disorders (SMQ)	Incubator therapy
Neonatal disorders (SMQ)	Apgar score abnormal
Neonatal disorders (SMQ)	Neonatal hypoparathyroidism
Neonatal disorders (SMQ)	Cleft lip repair
Neonatal disorders (SMQ)	Meconium stain
Neonatal disorders (SMQ)	Neonatal tetany
Neonatal disorders (SMQ)	Meconium plug syndrome
Neonatal disorders (SMQ)	Neonatal tachypnoea
Neonatal disorders (SMQ)	Meconium peritonitis
Neonatal disorders (SMQ)	Cranial sutures widening
Neonatal disorders (SMQ)	Poor sucking reflex
Neonatal disorders (SMQ)	Meningitis neonatal
Neonatal disorders (SMQ)	Neonatal intestinal dilatation
Neonatal disorders (SMQ)	Neonatal aspiration
Neonatal disorders (SMQ)	Overfeeding of infant
Neonatal disorders (SMQ)	Coma neonatal
Neonatal disorders (SMQ)	Head circumference abnormal
Neonatal disorders (SMQ)	Polycythaemia neonatorum

Neonatal disorders (SMQ)	Alloimmunisation
Neonatal disorders (SMQ)	Neonatal infection
Neonatal disorders (SMQ)	Oedema neonatal
Neonatal disorders (SMQ)	Respiratory disorder neonatal
Neonatal disorders (SMQ)	Neonatal complications of substance abuse
Neonatal disorders (SMQ)	Haemorrhage neonatal
Neonatal disorders (SMQ)	Infantile spitting up
Neonatal disorders (SMQ)	Rhesus incompatibility
Neonatal disorders (SMQ)	Balloon atrial septostomy
Neonatal disorders (SMQ)	Funisitis
Neonatal disorders (SMQ)	Venous thrombosis neonatal
Neonatal disorders (SMQ)	Thymus enlargement
Neonatal disorders (SMQ)	Arterial switch operation
Neonatal disorders (SMQ)	Aorticopulmonary window repair
Neonatal disorders (SMQ)	Tetralogy of Fallot repair
Neonatal disorders (SMQ)	Low birth weight baby
Neonatal disorders (SMQ)	Umbilical granuloma
Neonatal disorders (SMQ)	Pulmonary air leakage
Neonatal disorders (SMQ)	Norwood procedure
Neonatal disorders (SMQ)	Atrial switch operation
Neonatal disorders (SMQ)	Discordant twin
Neonatal disorders (SMQ)	Gasping syndrome
Neonatal disorders (SMQ)	Subgaleal haematoma
Neonatal disorders (SMQ)	Perinatal HIV infection
Neonatal disorders (SMQ)	Growth failure
Neonatal disorders (SMQ)	Maternal exposure during delivery
Neonatal disorders (SMQ)	Early infantile epileptic encephalopathy with burst-suppression
Neonatal disorders (SMQ)	Breast milk substitute intolerance
Neonatal disorders (SMQ)	Umbilical discharge
Neonatal disorders (SMQ)	Neonatal behavioural syndrome
Neonatal disorders (SMQ)	Transient neonatal pustular melanosis
Neonatal disorders (SMQ)	Neuroendocrine cell hyperplasia of infancy
Neonatal disorders (SMQ)	Pulmonary lymphangiectasia
Neonatal disorders (SMQ)	Developmental hip dysplasia
Neonatal disorders (SMQ)	Lenticulostriatal vasculopathy
Neonatal disorders (SMQ)	Perinatal stroke
Neonatal disorders (SMQ)	Parenteral nutrition associated liver disease
Neonatal disorders (SMQ)	Disturbance of thermoregulation of newborn

Neonatal disorders (SMQ)	Neonatal gastrointestinal haemorrhage
Neonatal disorders (SMQ)	Neonatal intestinal perforation
Neonatal disorders (SMQ)	Dry lung syndrome
Neonatal disorders (SMQ)	Meconium cyst
Neonatal disorders (SMQ)	Fixed bowel loop
Neonatal disorders (SMQ)	Junctional ectopic tachycardia
Neonatal disorders (SMQ)	Johanson-Blizzard syndrome
Neonatal disorders (SMQ)	Hypoplastic nasal cartilage
Neonatal disorders (SMQ)	Neonatal alloimmune thrombocytopenia
Neonatal disorders (SMQ)	Fryns syndrome
Neonatal disorders (SMQ)	Perinatal HBV infection
Neonatal disorders (SMQ)	Infantile vomiting
Neonatal disorders (SMQ)	Poor feeding infant
Neonatal disorders (SMQ)	Infantile haemangioma
Neonatal disorders (SMQ)	Neonatal testicular torsion
Neonatal disorders (SMQ)	Feeding intolerance
Neonatal disorders (SMQ)	Neonatal gastrointestinal disorder
Neonatal disorders (SMQ)	Meconium aspiration syndrome
Neonatal disorders (SMQ)	Primary familial hypomagnesaemia
Neonatal disorders (SMQ)	Periventricular haemorrhage neonatal
Neonatal disorders (SMQ)	Alveolar capillary dysplasia
Neonatal disorders (SMQ)	Infantile back arching
Neonatal disorders (SMQ)	Infantile apnoea
Neonatal disorders (SMQ)	Epileptic encephalopathy
Neonatal disorders (SMQ)	Familial infantile bilateral striatal necrosis
Neonatal disorders (SMQ)	Sporadic infantile bilateral striatal necrosis
Neonatal disorders (SMQ)	Neonatal haemochromatosis
Neonatal disorders (SMQ)	Neonatal toxicity
Neonatal disorders (SMQ)	Neonatal hypocalcaemia
Neonatal disorders (SMQ)	Posthaemorrhagic hydrocephalus
Neonatal disorders (SMQ)	Neonatal deafness
Neonatal disorders (SMQ)	Subgaleal haemorrhage
Neonatal disorders (SMQ)	Neonatal hypoacusis
Neonatal disorders (SMQ)	Birth defect correction
Neonatal disorders (SMQ)	Truncus arteriosus repair
Neonatal disorders (SMQ)	Neonatal bradyarrhythmia
Neonatal disorders (SMQ)	Neonatal tachyarrhythmia
Neonatal disorders (SMQ)	Neonatal pneumothorax
Neonatal disorders (SMQ)	Neonatal seizure
Neonatal disorders (SMQ)	Neonatal epileptic seizure

Neonatal disorders (SMQ)	Infant sedation
Neonatal disorders (SMQ)	Neonatal sinus bradycardia
Neonatal disorders (SMQ)	Infant irritability
Neonatal disorders (SMQ)	Constipation neonatal
Neonatal disorders (SMQ)	Neonatal sinus tachycardia
Neonatal disorders (SMQ)	Administration site reaction neonatal
Neonatal disorders (SMQ)	Pulmonary haemorrhage neonatal
Neonatal disorders (SMQ)	Infant dyschezia
Neonatal disorders (SMQ)	Small fontanelle
Neonatal disorders (SMQ)	Neonatal deformity
Neonatal disorders (SMQ)	Infantile acropustulosis
Neonatal disorders (SMQ)	Postnatal growth restriction
Neonatal disorders (SMQ)	Neonatal Crohn's disease
Neonatal disorders (SMQ)	Neonatal dyspnoea
Neonatal disorders (SMQ)	Midline head position
Neonatal disorders (SMQ)	Finnegan score increased
Neonatal disorders (SMQ)	Brief resolved unexplained event
Neonatal disorders (SMQ)	Delayed umbilical cord clamping
Neonatal disorders (SMQ)	Suck-swallow breathing coordination disturbance
Neonatal disorders (SMQ)	Neonatal hyperglycaemia
Neonatal disorders (SMQ)	Neonatal bacteraemia
Neonatal disorders (SMQ)	Scalp injury due to birth trauma
Neonatal disorders (SMQ)	Hypoxic ischaemic encephalopathy neonatal
Neonatal disorders (SMQ)	Intracranial haemorrhage neonatal
Neonatal disorders (SMQ)	Infantile postural asymmetry
Neonatal disorders (SMQ)	Subaponeurotic cerebrospinal fluid collection of infancy
Neonatal disorders (SMQ)	Neonatal bacterial pneumonia
Neonatal disorders (SMQ)	Transient neonatal hyperthyrotropinaemia
Neonatal disorders (SMQ)	High risk infant
Neonatal disorders (SMQ)	Delayed passage of meconium
Neonatal disorders (SMQ)	Infantile diarrhoea
Neonatal disorders (SMQ)	Gastrooesophageal reflux in neonate
Neonatal disorders (SMQ)	Neonatal pulmonary hypertension
Pregnancy, labour and delivery complications and risk factors (excl abortions and stillbirth) (SMQ)	Abnormal labour
Pregnancy, labour and delivery complications and risk factors (excl abortions and stillbirth) (SMQ)	Abnormal labour affecting foetus
Pregnancy, labour and delivery complications and risk factors (excl abortions and stillbirth) (SMQ)	Acute fatty liver of pregnancy

Pregnancy, labour and delivery complications and risk factors (excl abortions and stillbirth) (SMQ)	Amniocentesis abnormal
Pregnancy, labour and delivery complications and risk factors (excl abortions and stillbirth) (SMQ)	Amnioscopy abnormal
Pregnancy, labour and delivery complications and risk factors (excl abortions and stillbirth) (SMQ)	Antiphospholipid syndrome
Pregnancy, labour and delivery complications and risk factors (excl abortions and stillbirth) (SMQ)	Arrested labour
Pregnancy, labour and delivery complications and risk factors (excl abortions and stillbirth) (SMQ)	Artificial rupture of membranes
Pregnancy, labour and delivery complications and risk factors (excl abortions and stillbirth) (SMQ)	Bacteriuria in pregnancy
Pregnancy, labour and delivery complications and risk factors (excl abortions and stillbirth) (SMQ)	Benign hydatidiform mole
Pregnancy, labour and delivery complications and risk factors (excl abortions and stillbirth) (SMQ)	Breech delivery
Pregnancy, labour and delivery complications and risk factors (excl abortions and stillbirth) (SMQ)	Breech extraction
Pregnancy, labour and delivery complications and risk factors (excl abortions and stillbirth) (SMQ)	Breech presentation
Pregnancy, labour and delivery complications and risk factors (excl abortions and stillbirth) (SMQ)	Brief psychotic disorder, with postpartum onset
Pregnancy, labour and delivery complications and risk factors (excl abortions and stillbirth) (SMQ)	Caesarean section
Pregnancy, labour and delivery complications and risk factors (excl abortions and stillbirth) (SMQ)	Cephalo-pelvic disproportion
Pregnancy, labour and delivery complications and risk factors (excl abortions and stillbirth) (SMQ)	Cervical incompetence
Pregnancy, labour and delivery complications and risk factors (excl abortions and stillbirth) (SMQ)	Chloasma
Pregnancy, labour and delivery complications and risk factors (excl abortions and stillbirth) (SMQ)	Delayed delivery
Pregnancy, labour and delivery complications and risk factors (excl abortions and stillbirth) (SMQ)	Diabetes complicating pregnancy
Pregnancy, labour and delivery complications and risk factors (excl abortions and stillbirth) (SMQ)	Drug dependence, antepartum
Pregnancy, labour and delivery complications and risk factors (excl abortions and stillbirth) (SMQ)	Drug dependence, postpartum
Pregnancy, labour and delivery complications and risk factors (excl abortions and stillbirth) (SMQ)	Eclampsia
Pregnancy, labour and delivery complications and risk factors (excl abortions and stillbirth) (SMQ)	Ectopic pregnancy
Pregnancy, labour and delivery complications and risk factors (excl abortions and stillbirth) (SMQ)	Ectopic pregnancy termination
Pregnancy, labour and delivery complications and risk factors (excl abortions and stillbirth) (SMQ)	Elderly primigravida

Pregnancy, labour and delivery complications and risk factors (excl abortions and stillbirth) (SMQ)	Face presentation
Pregnancy, labour and delivery complications and risk factors (excl abortions and stillbirth) (SMQ)	Failed forceps delivery
Pregnancy, labour and delivery complications and risk factors (excl abortions and stillbirth) (SMQ)	Failed induction of labour
Pregnancy, labour and delivery complications and risk factors (excl abortions and stillbirth) (SMQ)	Failed trial of labour
Pregnancy, labour and delivery complications and risk factors (excl abortions and stillbirth) (SMQ)	False labour
Pregnancy, labour and delivery complications and risk factors (excl abortions and stillbirth) (SMQ)	Foetal arm prolapse
Pregnancy, labour and delivery complications and risk factors (excl abortions and stillbirth) (SMQ)	Foetal malposition
Pregnancy, labour and delivery complications and risk factors (excl abortions and stillbirth) (SMQ)	Forceps delivery
Pregnancy, labour and delivery complications and risk factors (excl abortions and stillbirth) (SMQ)	Gestational diabetes
Pregnancy, labour and delivery complications and risk factors (excl abortions and stillbirth) (SMQ)	Glucose tolerance impaired in pregnancy
Pregnancy, labour and delivery complications and risk factors (excl abortions and stillbirth) (SMQ)	Glycosuria during pregnancy
Pregnancy, labour and delivery complications and risk factors (excl abortions and stillbirth) (SMQ)	Haemorrhage in pregnancy
Pregnancy, labour and delivery complications and risk factors (excl abortions and stillbirth) (SMQ)	Herpes gestationis
Pregnancy, labour and delivery complications and risk factors (excl abortions and stillbirth) (SMQ)	High foetal head
Pregnancy, labour and delivery complications and risk factors (excl abortions and stillbirth) (SMQ)	Hyperemesis gravidarum
Pregnancy, labour and delivery complications and risk factors (excl abortions and stillbirth) (SMQ)	Impetigo herpetiformis
Pregnancy, labour and delivery complications and risk factors (excl abortions and stillbirth) (SMQ)	Incoordinate uterine action
Pregnancy, labour and delivery complications and risk factors (excl abortions and stillbirth) (SMQ)	Induced labour
Pregnancy, labour and delivery complications and risk factors (excl abortions and stillbirth) (SMQ)	Large for dates baby
Pregnancy, labour and delivery complications and risk factors (excl abortions and stillbirth) (SMQ)	Locked twins
Pregnancy, labour and delivery complications and risk factors (excl abortions and stillbirth) (SMQ)	Malignant hydatidiform mole
Pregnancy, labour and delivery complications and risk factors (excl abortions and stillbirth) (SMQ)	Malignant neoplasm of placenta
Pregnancy, labour and delivery complications and risk factors (excl abortions and stillbirth) (SMQ)	Mastitis postpartum

Pregnancy, labour and delivery complications and risk factors (excl abortions and stillbirth) (SMQ)	Morning sickness
Pregnancy, labour and delivery complications and risk factors (excl abortions and stillbirth) (SMQ)	Multiple pregnancy
Pregnancy, labour and delivery complications and risk factors (excl abortions and stillbirth) (SMQ)	Obstetrical pulmonary embolism
Pregnancy, labour and delivery complications and risk factors (excl abortions and stillbirth) (SMQ)	Obstructed labour
Pregnancy, labour and delivery complications and risk factors (excl abortions and stillbirth) (SMQ)	Oligohydramnios
Pregnancy, labour and delivery complications and risk factors (excl abortions and stillbirth) (SMQ)	Pelvic haematoma obstetric
Pregnancy, labour and delivery complications and risk factors (excl abortions and stillbirth) (SMQ)	Perineal haematoma
Pregnancy, labour and delivery complications and risk factors (excl abortions and stillbirth) (SMQ)	Perineal repair breakdown
Pregnancy, labour and delivery complications and risk factors (excl abortions and stillbirth) (SMQ)	Pituitary infarction
Pregnancy, labour and delivery complications and risk factors (excl abortions and stillbirth) (SMQ)	Placenta praevia
Pregnancy, labour and delivery complications and risk factors (excl abortions and stillbirth) (SMQ)	Placenta praevia haemorrhage
Pregnancy, labour and delivery complications and risk factors (excl abortions and stillbirth) (SMQ)	Placental disorder
Pregnancy, labour and delivery complications and risk factors (excl abortions and stillbirth) (SMQ)	Placental insufficiency
Pregnancy, labour and delivery complications and risk factors (excl abortions and stillbirth) (SMQ)	Placental necrosis
Pregnancy, labour and delivery complications and risk factors (excl abortions and stillbirth) (SMQ)	Placental polyp
Pregnancy, labour and delivery complications and risk factors (excl abortions and stillbirth) (SMQ)	Placental transfusion syndrome
Pregnancy, labour and delivery complications and risk factors (excl abortions and stillbirth) (SMQ)	Polyhydramnios
Pregnancy, labour and delivery complications and risk factors (excl abortions and stillbirth) (SMQ)	Postpartum hypopituitarism
Pregnancy, labour and delivery complications and risk factors (excl abortions and stillbirth) (SMQ)	Postpartum venous thrombosis
Pregnancy, labour and delivery complications and risk factors (excl abortions and stillbirth) (SMQ)	Postpartum haemorrhage
Pregnancy, labour and delivery complications and risk factors (excl abortions and stillbirth) (SMQ)	Postpartum neurosis
Pregnancy, labour and delivery complications and risk factors (excl abortions and stillbirth) (SMQ)	Postpartum sepsis
Pregnancy, labour and delivery complications and risk factors (excl abortions and stillbirth) (SMQ)	Postpartum uterine subinvolution

Pregnancy, labour and delivery complications and risk factors (excl abortions and stillbirth) (SMQ)	Pre-eclampsia
Pregnancy, labour and delivery complications and risk factors (excl abortions and stillbirth) (SMQ)	Precipitate labour
Pregnancy, labour and delivery complications and risk factors (excl abortions and stillbirth) (SMQ)	Pregnancy in habitual aborter
Pregnancy, labour and delivery complications and risk factors (excl abortions and stillbirth) (SMQ)	Pregnancy with advanced maternal age
Pregnancy, labour and delivery complications and risk factors (excl abortions and stillbirth) (SMQ)	Premature delivery
Pregnancy, labour and delivery complications and risk factors (excl abortions and stillbirth) (SMQ)	Premature labour
Pregnancy, labour and delivery complications and risk factors (excl abortions and stillbirth) (SMQ)	Premature rupture of membranes
Pregnancy, labour and delivery complications and risk factors (excl abortions and stillbirth) (SMQ)	Premature separation of placenta
Pregnancy, labour and delivery complications and risk factors (excl abortions and stillbirth) (SMQ)	Previous caesarean section
Pregnancy, labour and delivery complications and risk factors (excl abortions and stillbirth) (SMQ)	Prolonged labour
Pregnancy, labour and delivery complications and risk factors (excl abortions and stillbirth) (SMQ)	Prolonged pregnancy
Pregnancy, labour and delivery complications and risk factors (excl abortions and stillbirth) (SMQ)	Puerperal pyrexia
Pregnancy, labour and delivery complications and risk factors (excl abortions and stillbirth) (SMQ)	Retained placenta or membranes
Pregnancy, labour and delivery complications and risk factors (excl abortions and stillbirth) (SMQ)	Retained products of conception
Pregnancy, labour and delivery complications and risk factors (excl abortions and stillbirth) (SMQ)	Rubella in pregnancy
Pregnancy, labour and delivery complications and risk factors (excl abortions and stillbirth) (SMQ)	Shoulder dystocia
Pregnancy, labour and delivery complications and risk factors (excl abortions and stillbirth) (SMQ)	Third stage postpartum haemorrhage
Pregnancy, labour and delivery complications and risk factors (excl abortions and stillbirth) (SMQ)	Threatened labour
Pregnancy, labour and delivery complications and risk factors (excl abortions and stillbirth) (SMQ)	Transverse presentation
Pregnancy, labour and delivery complications and risk factors (excl abortions and stillbirth) (SMQ)	Traumatic delivery
Pregnancy, labour and delivery complications and risk factors (excl abortions and stillbirth) (SMQ)	Twin pregnancy
Pregnancy, labour and delivery complications and risk factors (excl abortions and stillbirth) (SMQ)	Ultrasound antenatal screen abnormal
Pregnancy, labour and delivery complications and risk factors (excl abortions and stillbirth) (SMQ)	Umbilical cord around neck

Pregnancy, labour and delivery complications and risk factors (excl abortions and stillbirth) (SMQ)	Umbilical cord compression
Pregnancy, labour and delivery complications and risk factors (excl abortions and stillbirth) (SMQ)	Umbilical cord prolapse
Pregnancy, labour and delivery complications and risk factors (excl abortions and stillbirth) (SMQ)	Umbilical cord short
Pregnancy, labour and delivery complications and risk factors (excl abortions and stillbirth) (SMQ)	Umbilical cord vascular disorder
Pregnancy, labour and delivery complications and risk factors (excl abortions and stillbirth) (SMQ)	Unstable foetal lie
Pregnancy, labour and delivery complications and risk factors (excl abortions and stillbirth) (SMQ)	Uterine atony
Pregnancy, labour and delivery complications and risk factors (excl abortions and stillbirth) (SMQ)	Uterine hypertonus
Pregnancy, labour and delivery complications and risk factors (excl abortions and stillbirth) (SMQ)	Uterine hypotonus
Pregnancy, labour and delivery complications and risk factors (excl abortions and stillbirth) (SMQ)	Uterine inversion
Pregnancy, labour and delivery complications and risk factors (excl abortions and stillbirth) (SMQ)	Uterine malposition
Pregnancy, labour and delivery complications and risk factors (excl abortions and stillbirth) (SMQ)	Uterine rupture
Pregnancy, labour and delivery complications and risk factors (excl abortions and stillbirth) (SMQ)	Vacuum extractor delivery
Pregnancy, labour and delivery complications and risk factors (excl abortions and stillbirth) (SMQ)	Vasa praevia
Pregnancy, labour and delivery complications and risk factors (excl abortions and stillbirth) (SMQ)	Ruptured ectopic pregnancy
Pregnancy, labour and delivery complications and risk factors (excl abortions and stillbirth) (SMQ)	Biopsy chorionic villous abnormal
Pregnancy, labour and delivery complications and risk factors (excl abortions and stillbirth) (SMQ)	Umbilical cord abnormality
Pregnancy, labour and delivery complications and risk factors (excl abortions and stillbirth) (SMQ)	Tocolysis
Pregnancy, labour and delivery complications and risk factors (excl abortions and stillbirth) (SMQ)	Cholestasis of pregnancy
Pregnancy, labour and delivery complications and risk factors (excl abortions and stillbirth) (SMQ)	HELLP syndrome
Pregnancy, labour and delivery complications and risk factors (excl abortions and stillbirth) (SMQ)	Peripartum cardiomyopathy
Pregnancy, labour and delivery complications and risk factors (excl abortions and stillbirth) (SMQ)	Gestational trophoblastic detachment
Pregnancy, labour and delivery complications and risk factors (excl abortions and stillbirth) (SMQ)	Afterbirth pain
Pregnancy, labour and delivery complications and risk factors (excl abortions and stillbirth) (SMQ)	Metastases to placenta

Pregnancy, labour and delivery complications and risk factors (excl abortions and stillbirth) (SMQ)	Uterine contractions during pregnancy
Pregnancy, labour and delivery complications and risk factors (excl abortions and stillbirth) (SMQ)	Amniotic infection syndrome of Blane
Pregnancy, labour and delivery complications and risk factors (excl abortions and stillbirth) (SMQ)	Amniorrhexis
Pregnancy, labour and delivery complications and risk factors (excl abortions and stillbirth) (SMQ)	High risk pregnancy
Pregnancy, labour and delivery complications and risk factors (excl abortions and stillbirth) (SMQ)	Brow presentation
Pregnancy, labour and delivery complications and risk factors (excl abortions and stillbirth) (SMQ)	Oblique presentation
Pregnancy, labour and delivery complications and risk factors (excl abortions and stillbirth) (SMQ)	Labour stimulation
Pregnancy, labour and delivery complications and risk factors (excl abortions and stillbirth) (SMQ)	Labour induction
Pregnancy, labour and delivery complications and risk factors (excl abortions and stillbirth) (SMQ)	Obstetric infection
Pregnancy, labour and delivery complications and risk factors (excl abortions and stillbirth) (SMQ)	Retroplacental haematoma
Pregnancy, labour and delivery complications and risk factors (excl abortions and stillbirth) (SMQ)	Placental dysplasia
Pregnancy, labour and delivery complications and risk factors (excl abortions and stillbirth) (SMQ)	Cervix dystocia
Pregnancy, labour and delivery complications and risk factors (excl abortions and stillbirth) (SMQ)	Cervix cerclage procedure
Pregnancy, labour and delivery complications and risk factors (excl abortions and stillbirth) (SMQ)	Intrauterine infection
Pregnancy, labour and delivery complications and risk factors (excl abortions and stillbirth) (SMQ)	Maternal distress during labour
Pregnancy, labour and delivery complications and risk factors (excl abortions and stillbirth) (SMQ)	Postpartum stress disorder
Pregnancy, labour and delivery complications and risk factors (excl abortions and stillbirth) (SMQ)	Renal disorder in pregnancy
Pregnancy, labour and delivery complications and risk factors (excl abortions and stillbirth) (SMQ)	Thyroid dysfunction in pregnancy
Pregnancy, labour and delivery complications and risk factors (excl abortions and stillbirth) (SMQ)	Placental chorioangioma
Pregnancy, labour and delivery complications and risk factors (excl abortions and stillbirth) (SMQ)	Foetal malpresentation
Pregnancy, labour and delivery complications and risk factors (excl abortions and stillbirth) (SMQ)	Maternal therapy to enhance foetal lung maturity
Pregnancy, labour and delivery complications and risk factors (excl abortions and stillbirth) (SMQ)	Missed labour
Pregnancy, labour and delivery complications and risk factors (excl abortions and stillbirth) (SMQ)	Amniotic cavity disorder

Pregnancy, labour and delivery complications and risk factors (excl abortions and stillbirth) (SMQ)	Amniotic cavity infection
Pregnancy, labour and delivery complications and risk factors (excl abortions and stillbirth) (SMQ)	Labour complication
Pregnancy, labour and delivery complications and risk factors (excl abortions and stillbirth) (SMQ)	Obstetric procedure complication
Pregnancy, labour and delivery complications and risk factors (excl abortions and stillbirth) (SMQ)	Placental neoplasm
Pregnancy, labour and delivery complications and risk factors (excl abortions and stillbirth) (SMQ)	Uterine contractions abnormal
Pregnancy, labour and delivery complications and risk factors (excl abortions and stillbirth) (SMQ)	Complication of pregnancy
Pregnancy, labour and delivery complications and risk factors (excl abortions and stillbirth) (SMQ)	Postpartum disorder
Pregnancy, labour and delivery complications and risk factors (excl abortions and stillbirth) (SMQ)	Assisted delivery
Pregnancy, labour and delivery complications and risk factors (excl abortions and stillbirth) (SMQ)	Complication of delivery
Pregnancy, labour and delivery complications and risk factors (excl abortions and stillbirth) (SMQ)	Placenta accreta
Pregnancy, labour and delivery complications and risk factors (excl abortions and stillbirth) (SMQ)	Amniotic fluid volume decreased
Pregnancy, labour and delivery complications and risk factors (excl abortions and stillbirth) (SMQ)	Amniotic fluid volume increased
Pregnancy, labour and delivery complications and risk factors (excl abortions and stillbirth) (SMQ)	Gestational oedema
Pregnancy, labour and delivery complications and risk factors (excl abortions and stillbirth) (SMQ)	Biochemical pregnancy
Pregnancy, labour and delivery complications and risk factors (excl abortions and stillbirth) (SMQ)	Rhesus incompatibility
Pregnancy, labour and delivery complications and risk factors (excl abortions and stillbirth) (SMQ)	Lymphocytic hypophysitis
Pregnancy, labour and delivery complications and risk factors (excl abortions and stillbirth) (SMQ)	Heterotopic pregnancy
Pregnancy, labour and delivery complications and risk factors (excl abortions and stillbirth) (SMQ)	Omphalorrhexis
Pregnancy, labour and delivery complications and risk factors (excl abortions and stillbirth) (SMQ)	Umbilical cord haemorrhage
Pregnancy, labour and delivery complications and risk factors (excl abortions and stillbirth) (SMQ)	Placental infarction
Pregnancy, labour and delivery complications and risk factors (excl abortions and stillbirth) (SMQ)	Symphysiotomy
Pregnancy, labour and delivery complications and risk factors (excl abortions and stillbirth) (SMQ)	Amniotic fluid erythropoietin level increased
Pregnancy, labour and delivery complications and risk factors (excl abortions and stillbirth) (SMQ)	Drug exposure before pregnancy

Pregnancy, labour and delivery complications and risk factors (excl abortions and stillbirth) (SMQ)	Prophylaxis of abortion
Pregnancy, labour and delivery complications and risk factors (excl abortions and stillbirth) (SMQ)	Maternal alcohol use
Pregnancy, labour and delivery complications and risk factors (excl abortions and stillbirth) (SMQ)	Varicose veins vaginal
Pregnancy, labour and delivery complications and risk factors (excl abortions and stillbirth) (SMQ)	Polymorphic eruption of pregnancy
Pregnancy, labour and delivery complications and risk factors (excl abortions and stillbirth) (SMQ)	Lochial infection
Pregnancy, labour and delivery complications and risk factors (excl abortions and stillbirth) (SMQ)	Uterine hyperstimulation
Pregnancy, labour and delivery complications and risk factors (excl abortions and stillbirth) (SMQ)	Prophylaxis against Rh isoimmunisation
Pregnancy, labour and delivery complications and risk factors (excl abortions and stillbirth) (SMQ)	Anaemia of pregnancy
Pregnancy, labour and delivery complications and risk factors (excl abortions and stillbirth) (SMQ)	Amniorrhoea
Pregnancy, labour and delivery complications and risk factors (excl abortions and stillbirth) (SMQ)	Anaphylactoid syndrome of pregnancy
Pregnancy, labour and delivery complications and risk factors (excl abortions and stillbirth) (SMQ)	Venous thrombosis in pregnancy
Pregnancy, labour and delivery complications and risk factors (excl abortions and stillbirth) (SMQ)	Amniotic fluid index abnormal
Pregnancy, labour and delivery complications and risk factors (excl abortions and stillbirth) (SMQ)	Tubal rupture
Pregnancy, labour and delivery complications and risk factors (excl abortions and stillbirth) (SMQ)	Intrapartum haemorrhage
Pregnancy, labour and delivery complications and risk factors (excl abortions and stillbirth) (SMQ)	Shortened cervix
Pregnancy, labour and delivery complications and risk factors (excl abortions and stillbirth) (SMQ)	Uterine cervix stenosis
Pregnancy, labour and delivery complications and risk factors (excl abortions and stillbirth) (SMQ)	Placental hypertrophy
Pregnancy, labour and delivery complications and risk factors (excl abortions and stillbirth) (SMQ)	Decidual cast
Pregnancy, labour and delivery complications and risk factors (excl abortions and stillbirth) (SMQ)	Mirror syndrome
Pregnancy, labour and delivery complications and risk factors (excl abortions and stillbirth) (SMQ)	Discordant twin
Pregnancy, labour and delivery complications and risk factors (excl abortions and stillbirth) (SMQ)	Prenatal screening test abnormal
Pregnancy, labour and delivery complications and risk factors (excl abortions and stillbirth) (SMQ)	Pregnancy with young maternal age
Pregnancy, labour and delivery complications and risk factors (excl abortions and stillbirth) (SMQ)	Uterine dehiscence

Pregnancy, labour and delivery complications and risk factors (excl abortions and stillbirth) (SMQ)	Gestational hypertension
Pregnancy, labour and delivery complications and risk factors (excl abortions and stillbirth) (SMQ)	External cephalic version
Pregnancy, labour and delivery complications and risk factors (excl abortions and stillbirth) (SMQ)	Inferior vena cava syndrome
Pregnancy, labour and delivery complications and risk factors (excl abortions and stillbirth) (SMQ)	Subchorionic haemorrhage
Pregnancy, labour and delivery complications and risk factors (excl abortions and stillbirth) (SMQ)	Vulvovaginal injury
Pregnancy, labour and delivery complications and risk factors (excl abortions and stillbirth) (SMQ)	Exposure via father
Pregnancy, labour and delivery complications and risk factors (excl abortions and stillbirth) (SMQ)	Foetal exposure during pregnancy
Pregnancy, labour and delivery complications and risk factors (excl abortions and stillbirth) (SMQ)	Foetal exposure timing unspecified
Pregnancy, labour and delivery complications and risk factors (excl abortions and stillbirth) (SMQ)	Maternal exposure before pregnancy
Pregnancy, labour and delivery complications and risk factors (excl abortions and stillbirth) (SMQ)	Maternal exposure during pregnancy
Pregnancy, labour and delivery complications and risk factors (excl abortions and stillbirth) (SMQ)	Foetal exposure during delivery
Pregnancy, labour and delivery complications and risk factors (excl abortions and stillbirth) (SMQ)	Maternal exposure timing unspecified
Pregnancy, labour and delivery complications and risk factors (excl abortions and stillbirth) (SMQ)	Foetal monitoring abnormal
Pregnancy, labour and delivery complications and risk factors (excl abortions and stillbirth) (SMQ)	Umbilical cord thrombosis
Pregnancy, labour and delivery complications and risk factors (excl abortions and stillbirth) (SMQ)	Small size placenta
Pregnancy, labour and delivery complications and risk factors (excl abortions and stillbirth) (SMQ)	Chronic villitis of unknown etiology
Pregnancy, labour and delivery complications and risk factors (excl abortions and stillbirth) (SMQ)	Delivery outside health facility
Pregnancy, labour and delivery complications and risk factors (excl abortions and stillbirth) (SMQ)	Kleihauer-Betke test positive
Pregnancy, labour and delivery complications and risk factors (excl abortions and stillbirth) (SMQ)	Subchorionic haematoma
Pregnancy, labour and delivery complications and risk factors (excl abortions and stillbirth) (SMQ)	Puerperal infection
Pregnancy, labour and delivery complications and risk factors (excl abortions and stillbirth) (SMQ)	Peripartum haemorrhage
Pregnancy, labour and delivery complications and risk factors (excl abortions and stillbirth) (SMQ)	Pregnancy of unknown location
Pregnancy, labour and delivery complications and risk factors (excl abortions and stillbirth) (SMQ)	Preterm premature rupture of membranes

Pregnancy, labour and delivery complications and risk factors (excl abortions and stillbirth) (SMQ)	Prolonged rupture of membranes
Pregnancy, labour and delivery complications and risk factors (excl abortions and stillbirth) (SMQ)	Induction of cervix ripening
Pregnancy, labour and delivery complications and risk factors (excl abortions and stillbirth) (SMQ)	Postponement of preterm delivery
Pregnancy, labour and delivery complications and risk factors (excl abortions and stillbirth) (SMQ)	Reversible cerebral vasoconstriction syndrome
Pregnancy, labour and delivery complications and risk factors (excl abortions and stillbirth) (SMQ)	Perineal injury
Pregnancy, labour and delivery complications and risk factors (excl abortions and stillbirth) (SMQ)	Exposure during pregnancy
Pregnancy, labour and delivery complications and risk factors (excl abortions and stillbirth) (SMQ)	Ectopic pregnancy under hormonal contraception
Pregnancy, labour and delivery complications and risk factors (excl abortions and stillbirth) (SMQ)	Uterine adhesions
Pregnancy, labour and delivery complications and risk factors (excl abortions and stillbirth) (SMQ)	Ectopic pregnancy with contraceptive device
Pregnancy, labour and delivery complications and risk factors (excl abortions and stillbirth) (SMQ)	Uterine scar
Pregnancy, labour and delivery complications and risk factors (excl abortions and stillbirth) (SMQ)	Endometritis bacterial
Pregnancy, labour and delivery complications and risk factors (excl abortions and stillbirth) (SMQ)	Hyperreactio luteinalis
Pregnancy, labour and delivery complications and risk factors (excl abortions and stillbirth) (SMQ)	Amniotic fluid index decreased
Pregnancy, labour and delivery complications and risk factors (excl abortions and stillbirth) (SMQ)	Amniotic fluid index increased
Pregnancy, labour and delivery complications and risk factors (excl abortions and stillbirth) (SMQ)	Uterine scar diverticulum
Pregnancy, labour and delivery complications and risk factors (excl abortions and stillbirth) (SMQ)	Postpartum thrombosis
Pregnancy, labour and delivery complications and risk factors (excl abortions and stillbirth) (SMQ)	Tracheloplasty
Pregnancy, labour and delivery complications and risk factors (excl abortions and stillbirth) (SMQ)	Retained placenta operation
Pregnancy, labour and delivery complications and risk factors (excl abortions and stillbirth) (SMQ)	Risk of future pregnancy miscarriage
Pregnancy, labour and delivery complications and risk factors (excl abortions and stillbirth) (SMQ)	Uterine compression sutures
Pregnancy, labour and delivery complications and risk factors (excl abortions and stillbirth) (SMQ)	Amniotic membrane rupture test positive
Pregnancy, labour and delivery complications and risk factors (excl abortions and stillbirth) (SMQ)	Blood type incompatibility
Pregnancy, labour and delivery complications and risk factors (excl abortions and stillbirth) (SMQ)	Silent thyroiditis

Pregnancy, labour and delivery complications and risk factors (excl abortions and stillbirth) (SMQ)	Umbilical cord cyst
Pregnancy, labour and delivery complications and risk factors (excl abortions and stillbirth) (SMQ)	Uterine irritability
Pregnancy, labour and delivery complications and risk factors (excl abortions and stillbirth) (SMQ)	Short interpregnancy interval
Pregnancy, labour and delivery complications and risk factors (excl abortions and stillbirth) (SMQ)	Drug use disorder, antepartum
Pregnancy, labour and delivery complications and risk factors (excl abortions and stillbirth) (SMQ)	Drug use disorder, postpartum
Pregnancy, labour and delivery complications and risk factors (excl abortions and stillbirth) (SMQ)	Angiotensin II receptor type 1 antibody positive
Pregnancy, labour and delivery complications and risk factors (excl abortions and stillbirth) (SMQ)	Labour augmentation
Pregnancy, labour and delivery complications and risk factors (excl abortions and stillbirth) (SMQ)	Maternal cancer in pregnancy
Pregnancy, labour and delivery complications and risk factors (excl abortions and stillbirth) (SMQ)	Uterine tachysystole
Pregnancy, labour and delivery complications and risk factors (excl abortions and stillbirth) (SMQ)	Paternal exposure during pregnancy
Pregnancy, labour and delivery complications and risk factors (excl abortions and stillbirth) (SMQ)	Paternal exposure timing unspecified
Pregnancy, labour and delivery complications and risk factors (excl abortions and stillbirth) (SMQ)	Paternal exposure before pregnancy
Pregnancy, labour and delivery complications and risk factors (excl abortions and stillbirth) (SMQ)	Chorioamniotic separation
Pregnancy, labour and delivery complications and risk factors (excl abortions and stillbirth) (SMQ)	Threatened uterine rupture
Pregnancy, labour and delivery complications and risk factors (excl abortions and stillbirth) (SMQ)	Bimanual uterine compression
Pregnancy, labour and delivery complications and risk factors (excl abortions and stillbirth) (SMQ)	Placental lake
Pregnancy, labour and delivery complications and risk factors (excl abortions and stillbirth) (SMQ)	Placental calcification
Pregnancy, labour and delivery complications and risk factors (excl abortions and stillbirth) (SMQ)	Uterine hypokinesia
Pregnancy, labour and delivery complications and risk factors (excl abortions and stillbirth) (SMQ)	Placenta duplex
Pregnancy, labour and delivery complications and risk factors (excl abortions and stillbirth) (SMQ)	Postpartum anxiety
Pregnancy, labour and delivery complications and risk factors (excl abortions and stillbirth) (SMQ)	Asynclitic presentation
Pregnancy, labour and delivery complications and risk factors (excl abortions and stillbirth) (SMQ)	Placental cyst
Pregnancy, labour and delivery complications and risk factors (excl abortions and stillbirth) (SMQ)	Pelvic girdle pain

Pregnancy, labour and delivery complications and risk factors (excl abortions and stillbirth) (SMQ)	Foetal dystocia
Pregnancy, labour and delivery complications and risk factors (excl abortions and stillbirth) (SMQ)	Umbilical artery vascular resistance increased
Pregnancy, labour and delivery complications and risk factors (excl abortions and stillbirth) (SMQ)	Superimposed pre-eclampsia
Pregnancy, labour and delivery complications and risk factors (excl abortions and stillbirth) (SMQ)	Abnormal cord insertion
Pregnancy, labour and delivery complications and risk factors (excl abortions and stillbirth) (SMQ)	Uterine diverticulum
Pregnancy, labour and delivery complications and risk factors (excl abortions and stillbirth) (SMQ)	Maternal exposure via partner during pregnancy
Pregnancy, labour and delivery complications and risk factors (excl abortions and stillbirth) (SMQ)	Cervix scarring
Pregnancy, labour and delivery complications and risk factors (excl abortions and stillbirth) (SMQ)	Foetal vascular malperfusion
Pregnancy, labour and delivery complications and risk factors (excl abortions and stillbirth) (SMQ)	Lochiostasis
Pregnancy, labour and delivery complications and risk factors (excl abortions and stillbirth) (SMQ)	Uterine artery embolisation
Pregnancy, labour and delivery complications and risk factors (excl abortions and stillbirth) (SMQ)	Genital scarring
Pregnancy, labour and delivery complications and risk factors (excl abortions and stillbirth) (SMQ)	Vaginal tamponade
Pregnancy, labour and delivery complications and risk factors (excl abortions and stillbirth) (SMQ)	Reproductive tract procedural infection
Pregnancy, labour and delivery complications and risk factors (excl abortions and stillbirth) (SMQ)	Perineal swelling
Pregnancy, labour and delivery complications and risk factors (excl abortions and stillbirth) (SMQ)	Maternal death
Pregnancy, labour and delivery complications and risk factors (excl abortions and stillbirth) (SMQ)	Obstetrical tetanus
Pregnancy, labour and delivery complications and risk factors (excl abortions and stillbirth) (SMQ)	Pregnancy related infection
Pregnancy, labour and delivery complications and risk factors (excl abortions and stillbirth) (SMQ)	Medically induced preterm birth
Pregnancy, labour and delivery complications and risk factors (excl abortions and stillbirth) (SMQ)	Maternal body mass index decreased
Pregnancy, labour and delivery complications and risk factors (excl abortions and stillbirth) (SMQ)	Maternal body mass index increased
Pregnancy, labour and delivery complications and risk factors (excl abortions and stillbirth) (SMQ)	Excessive maternal gestational weight gain
Pregnancy, labour and delivery complications and risk factors (excl abortions and stillbirth) (SMQ)	Maternal pre-pregnancy underweight
Pregnancy, labour and delivery complications and risk factors (excl abortions and stillbirth) (SMQ)	Maternal pre-pregnancy obesity

Pregnancy, labour and delivery complications and risk factors (excl abortions and stillbirth) (SMQ)	Placental oedema
Pregnancy, labour and delivery complications and risk factors (excl abortions and stillbirth) (SMQ)	Prurigo of pregnancy
Pregnancy, labour and delivery complications and risk factors (excl abortions and stillbirth) (SMQ)	Maternal disease complicating pregnancy
Termination of pregnancy and risk of abortion (SMQ)	Aborted pregnancy
Termination of pregnancy and risk of abortion (SMQ)	Abortion
Termination of pregnancy and risk of abortion (SMQ)	Abortion complete complicated
Termination of pregnancy and risk of abortion (SMQ)	Abortion incomplete
Termination of pregnancy and risk of abortion (SMQ)	Abortion incomplete complicated
Termination of pregnancy and risk of abortion (SMQ)	Abortion induced
Termination of pregnancy and risk of abortion (SMQ)	Abortion induced complete complicated
Termination of pregnancy and risk of abortion (SMQ)	Abortion induced complicated
Termination of pregnancy and risk of abortion (SMQ)	Abortion induced incomplete complicated
Termination of pregnancy and risk of abortion (SMQ)	Abortion infected
Termination of pregnancy and risk of abortion (SMQ)	Abortion missed
Termination of pregnancy and risk of abortion (SMQ)	Abortion spontaneous
Termination of pregnancy and risk of abortion (SMQ)	Abortion spontaneous complete complicated
Termination of pregnancy and risk of abortion (SMQ)	Abortion spontaneous complicated
Termination of pregnancy and risk of abortion (SMQ)	Abortion spontaneous incomplete complicated
Termination of pregnancy and risk of abortion (SMQ)	Abortion threatened
Termination of pregnancy and risk of abortion (SMQ)	Ectopic pregnancy termination
Termination of pregnancy and risk of abortion (SMQ)	Evacuation of retained products of conception
Termination of pregnancy and risk of abortion (SMQ)	Mycoplasmal postabortal fever
Termination of pregnancy and risk of abortion (SMQ)	Post abortion complication
Termination of pregnancy and risk of abortion (SMQ)	Post abortion haemorrhage
Termination of pregnancy and risk of abortion (SMQ)	Retained products of conception
Termination of pregnancy and risk of abortion (SMQ)	Stillbirth
Termination of pregnancy and risk of abortion (SMQ)	Imminent abortion
Termination of pregnancy and risk of abortion (SMQ)	Induced abortion haemorrhage
Termination of pregnancy and risk of abortion (SMQ)	Induced abortion infection
Termination of pregnancy and risk of abortion (SMQ)	Abortion early
Termination of pregnancy and risk of abortion (SMQ)	Abortion late
Termination of pregnancy and risk of abortion (SMQ)	Induced abortion failed
Termination of pregnancy and risk of abortion (SMQ)	Abortion induced incomplete
Termination of pregnancy and risk of abortion (SMQ)	Foetal death
Termination of pregnancy and risk of abortion (SMQ)	Abnormal product of conception
Termination of pregnancy and risk of abortion (SMQ)	Abortion induced complete

Termination of pregnancy and risk of abortion (SMQ)	Post abortion infection
Termination of pregnancy and risk of abortion (SMQ)	Abortion complete
Termination of pregnancy and risk of abortion (SMQ)	Abortion complicated
Termination of pregnancy and risk of abortion (SMQ)	Abortion spontaneous complete
Termination of pregnancy and risk of abortion (SMQ)	Abortion spontaneous incomplete
Termination of pregnancy and risk of abortion (SMQ)	Habitual abortion
Termination of pregnancy and risk of abortion (SMQ)	Biochemical pregnancy
Termination of pregnancy and risk of abortion (SMQ)	Prophylaxis of abortion
Termination of pregnancy and risk of abortion (SMQ)	Molar abortion
Termination of pregnancy and risk of abortion (SMQ)	Abortion of ectopic pregnancy
Termination of pregnancy and risk of abortion (SMQ)	Selective abortion
Termination of pregnancy and risk of abortion (SMQ)	Vanishing twin syndrome
Termination of pregnancy and risk of abortion (SMQ)	Twin reversed arterial perfusion sequence malformation
Termination of pregnancy and risk of abortion (SMQ)	Foeticide
Termination of pregnancy and risk of abortion (SMQ)	Premature baby death
Termination of pregnancy and risk of abortion (SMQ)	Risk of future pregnancy miscarriage
Termination of pregnancy and risk of abortion (SMQ)	Anembryonic gestation
Termination of pregnancy and risk of abortion (SMQ)	Lithopedion
Termination of pregnancy and risk of abortion (SMQ)	Vacuum aspiration
