# Supplementary material for: Pregnancy Recommendations Solely Based on Preclinical Evidence Should Be Integrated with Real-World Evidence: A Disproportionality Analysis of Certolizumab and Other TNF-Alpha Inhibitors Used in Pregnant Patients with Psoriasis
Source: Pharmaceuticals (Basel). 2024 Jul 7;17(7):904. doi: 10.3390/ph17070904 (PMC11279583; doi:10.3390/ph17070904)
Supplement: Supplementary file 1 [file pharmaceuticals-17-00904-s001.zip › Supplementary Table S2.pdf]

Table S2. Characteristics of Individual Case Safety Reports (ICSRs) for non-pregnant women with psoriasis, stratified by each anti-TNF drugs.

	**Anti-TNF drugs**				
	**Adalimumab**	**Certolizumab**	**Etanercept**	**Golimumab**	**Infliximab**
**N. of ICSRs**	4,493	803	4,614	269	1,602
**Age range (%)**					
0-1 Month	0 (0.0)	2 (0.2)	2 (<0.1)	0 (0.0)	0 (0.0)
2 Months - 2 Years	0 (0.0)	0 (0.0)	1 (< 0.1)	0 (0.0)	0 (0.0)
3-11 Years	21 (0.5)	0 (0.0)	17 (0.4)	2 (0.7)	2 (0.1)
12-17 Years	33 (0.7)	0 (0.0)	51 (1.1)	0 (0.0)	7 (0.4)
18-64 Years	2,830 (63.0)	503 (62.6)	3,050 (66.1)	174 (64.7)	1,124 (70.2)
65-85 Years	652 (14.5)	44 (5.5)	974 (21.1)	10 (3.7)	209 (13.0)
More than 85 Years	11 (0.2)	1 (0.1)	31 (0.7)	0 (0.0)	5 (0.3)
Not Specified	946 (21.1)	253 (31.5)	488 (10.6)	83 (30.9)	255 (15.9)
**Type of report = Spontaneous (%)**	4,493 (100.0)	803 (100.0)	4,614 (100.0)	269 (100.0)	1,602 (100.0)
**Source qualification (%)**					
Healthcare Professional	3,608 (80.3)	620 (77.2)	2,481 (53.8)	230 (85.5)	1,404 (87.6)
Non-Healthcare Professional	884 (19.7)	183 (22.8)	2,133 (46.2)	39 (14.5)	198 (12.4)
Not Specified	1 (< 0.1)	0 (0.0)	0 (0.0)	0 (0.0)	0 (0.0)
