# Supplementary material for: Pregnancy Recommendations Solely Based on Preclinical Evidence Should Be Integrated with Real-World Evidence: A Disproportionality Analysis of Certolizumab and Other TNF-Alpha Inhibitors Used in Pregnant Patients with Psoriasis
Source: Pharmaceuticals (Basel). 2024 Jul 7;17(7):904. doi: 10.3390/ph17070904 (PMC11279583; doi:10.3390/ph17070904)
Supplement: Supplementary file 1 [file pharmaceuticals-17-00904-s001.zip › Supplementary Table S3.pdf]

Table S3. Comparison of adverse events between pregnant and non-pregnant women, including cases where the indication for anti-TNF alpha treatment is not specified, by comparing the odds of reporting events (categorized by System Organ Class) with certolizumab versus other anti-TNF-drugs.

	**Certolizumab vs. other TNF-alfa drugs (ROR (95% CI))**	
**System Organ Class**	**Pregnant population**	**Non-pregnant population**
Blood and lymphatic system disorders	(less than 3 cases)	0.55 (0.44–0.70)
Cardiac disorders	1.00 (0.80–1.25)	0.90 (0.77–1.04)
Congenital, familial and genetic disorders	2.07 (0.87–4.94)	0.92 (0.70–1.21)
Ear and labyrinth disorders	(less than 3 cases)	1.09 (0.82–1.44)
Endocrine disorders	(less than 3 cases)	0.99 (0.65–1.51)
Eye disorders	(less than 3 cases)	0.71 (0.60–0.84)
Gastrointestinal disorders	0.94 (0.84–1.05)	1.12 (1.04–1.21)
General disorders	0.97 (0.90–1.04)	1.02 (0.97–1.07)
Hepatobiliary disorders	0.82 (0.52–1.30)	0.73 (0.59–0.91)
Immune system disorders	1.01 (0.83–1.22)	0.92 (0.81–1.04)
Infections and infestations	1.00 (0.82–1.23)	0.77 (0.73–0.82)
Injury, poisoning and procedural complications	1.12 (1.03–1.21)	1.29 (1.22–1.37)
Investigations	0.94 (0.83–1.06)	0.70 (0.63–0.78)
Metabolism and nutrition disorders	0.99 (0.77–1.29)	0.71 (0.57–0.88)
Musculoskeletal and connective tissue disorders	0.97 (0.90–1.05)	0.92 (0.87–0.99)
Neoplasms benign, malignant and unspecified	1.03 (0.42–2.56)	0.53 (0.47–0.61)
Nervous system disorders	0.95 (0.79–1.15)	0.98 (0.90–1.06)
Pregnancy, puerperium and perinatal conditions	1.30 (1.02–1.65)	1.91 (1.63–2.23)
Product issues	(less than 3 cases)	2.69 (2.40–3.01)
Psychiatric disorders	0.96 (0.63–1.47)	0.97 (0.84–1.11)
Renal and urinary disorders	(less than 3 cases)	0.71 (0.56–0.89)
Reproductive system and breast disorders	2.41 (0.68–8.56)	0.99 (0.76–1.29)
Respiratory, thoracic and mediastinal disorders	1.05 (0.70–1.59)	0.73 (0.65–0.81)
Skin and subcutaneous tissue disorders	0.98 (0.88–1.09)	1.40 (1.32–1.49)
Social circumstances	1.45 (0.64–3.29)	1.49 (1.16–1.91)
Surgical and medical procedures	1.15 (0.87–1.52)	1.98 (1.77–2.20)
Vascular disorders	0.99 (0.78–1.27)	0.65 (0.55–0.76)
